
JOURNAL INFORMATION
==============================
NLM Title Abbreviation: Thyroid Res
Journal ID: Thyroid Res
NLM Journal ID: 101469037

Thyroid Research

EISSN: 1756-6614
Publisher: BMC

ARTICLE INFORMATION
==============================
PMCID: PMC5606214
DOI: 10.1186/s13044-017-0040-7
Article ID: 40
Article version: 1
Subjects: Meeting Abstracts

Meeting abstracts from the 65th British Thyroid Association Annual Meeting

London, UK. 16 May 2017

Electronic publication date: 2017 Sep 19
Publication date: 2017
Volume: 10
Issue: Suppl 2
Issue sponsor: The publication of this supplement has been funded by the British Thyroid Association.
Electronic Location ID: 5
Copyright: © The Author(s). 2017
License: Open AccessThis article is distributed under the terms of the Creative Commons Attribution 4.0 International License (http://creativecommons.org/licenses/by/4.0/), which permits unrestricted use, distribution, and reproduction in any medium, provided you give appropriate credit to the original author(s) and the source, provide a link to the Creative Commons license, and indicate if changes were made. The Creative Commons Public Domain Dedication waiver (http://creativecommons.org/publicdomain/zero/1.0/) applies to the data made available in this article, unless otherwise stated.
License URL: https://creativecommons.org/licenses/by/4.0/

Conference name: 65th British Thyroid Association Annual Meeting
Conference Acronym: BTA 2017
Conference location: London, UK
Conference date: 16 May 2017
issue-copyright-statement: © The Author(s) 2017

==============================
Download to read this full article text

Publisher’s Note

Springer Nature remains neutral with regard to jurisdictional claims in published maps and institutional affiliations.

